# The Diagnostic Value of the Urine

**Published:** 1850-03

**Authors:** E. B. Haskins


### WESTERN JOURNAL

OF

### MEDICINE AND SURGERY.

MARCH, 1850.

Art. I.—*The Diagnostic Value of the Urine.* A Paper read before a meeting of the Montgomery Medical Society, held at Clarksville, Tenn., Nov. 3, 1849. By E. B. Haskins. Published by order of the Society.

Within the last few years, many valuable aids have been furnished the medical practitioner in rendering his diagnosis more certain, and, consequently, his treatment more rational and efficient: amongst the most important of which may be mentioned, auscultation, mediate and immediate; utero-vaginal exploration with the speculum, and physico-chemical observations upon the blood and urine.

These three important diagnostic agents, having been directed to different departments of semeiological investigations, extend almost over the entire field of pathological inquiry. Auscultution and utero-vaginal exploration seem to be fully understood and appreciated by the profession at large. So much so, that it is seldom a medical man is met with, who has not, either at home or in his pocket, both a stethoscope and a speculum. The same, however, cannot be affirmed of the other means of diagnosis—the test-tube and gravemeter, though equally valuable, equally portable, and of equal applicability, are seldom or never to be seen. It is this comparative neglect, by the profession generally, of so important a key to the seat and nature of morbid actions, that has determined me to discharge my obligation, on the present occasion, by offering some reflections upon the diagnostic value of the urine.

In order to a just conception, and proper appreciation of the relations of the renal secretion to *morbid* actions, it is necessary first to take a view of the sources and nature of that secretion, and its relations to *healthy* actions. Such a view, however, in the narrow limits assigned to this paper, can only be *general*, and imperfect in detail.

The entire bulk of ingested matter, water excepted, that is digested and absorbed into the circulation of man and the higher order of animals, belongs to one or the other of two great divisions of alimentary substances—nitrogenous, and carbonaceous—and each is destined to ultimate solution in the organism in subservience of the ends of organic and animal life. The former, or nitrogenous substances, first enter into *vital* connection with the living tissues, and subsequently dissolve in giving rise to certain phenomena, as muscular contraction, and the various active demonstrations of the brain and nervous system; the latter, or carbonaceous, enter directly into co[illegible]bination with the oxygen of the inspired air, giving rise, in the course of the circulation, to the phenomenon of animal heat. Along with these substances, certain minerals enter the circulation for certain ends not necessary to mention here. Notwithstanding this is the destiny of the larger portion of the absorbed alimentary matter; yet there is a portion of both the nitrogenous and carbonaceous that is unappropriated, and is, therefore, cast out of the system, along with the decayed matter, as superfluous.

Now, these two great classes of effete and superfluous substances, corresponding in chemical constitution with the absorbed materials, are respectively provided with eliminating or depurating organs: the nitrogenous, with the skin and kidneys, and the carbonaceous with the lungs and liver.

But these alimentary substances do not pass out of the economy in the same form in which they entered it. The carbonaceous, as oils, starch, gum, and sugar, are mostly reduced in the organism to carbonic acid and water, which escape mainly by the lungs, and that which is not thus reduced, (or, for a time deposited as fat in the adipose tissue) being eliminated by the liver in the form of fatty acids, in combination with alkaline bases. The nitrogenous substances, as albumen, fibrin, &c., on the other hand, are, for the most part, eliminated by the kidneys in the form of urea, uric and hippuric acids, &c. There is every reason to believe, also, that some portion of the effete nitrogenous substances pass off by the skin. Nearly all of the water entering the system, as well as that which is generated in the economy, finds egress by the skin, lungs, and kidneys, respectively.

It will now be seen at a single glance, that these depurating organs are closely and constantly related to every tissue in the animal body, and thus their functional products are alike related to every vital as well as chemical action in the organism. This being the case, then, it must be obvious, that, independent of observations, any deviation from a physiological state in the organs of assimilation, must produce a corresponding deviation in those of secretion and excretion.

The exhalations of the *skin* and *lungs*, for the want of means of readily collecting a sufficient quantity for analysis, can never be of great value for diagnostic purposes. The *bile*, as it reaches the eye of the observer, having traversed the entire length of the intestinal tube, being mixed up with the secretions of the mucous follicles, the gastric and pancreatic juices, the remains of various kinds of aliments, and with gaseous products—can, at best, only furnish a clue to the degree of activity of the hepatic function.

The *urine*, however, is clear of all those objections and difficulties. It is secreted and emptied into a capacious receptacle, and there remains, free from material change, until enough is collected for analytical purposes; and the passage, through which it is discharged, is short and free from foreign matter.* But we are not left to *a priori* reasoning, to establish the *diagnostic value of the urine*—we have observation and experience as ample upon this subject as any other in the whole scope of medical science. There has, perhaps, no class of diseases been more fully and satisfactorily elucidated, and by more able and trustworthy men, than that class which makes a notable impress upon the renal secretion; and from the increasing interest given to physiology and pathology, by the agency of chemico-microscopy, no class of diseases presents a more hopeful prospective view.

Then, in order to present in the strongest light, the claims of the urine to the careful inspection of the diagnostician, I propose, hastily, to consider the most prominent of those diseases under two general divisions:

1. *Diseases that cannot be diagnosed without the aid of the urine.*

---

* Menstrual fluid and vaginal mucus may, sometimes, produce deception in the urine of females; but with a little care they are easily avoided.

2. *Diseases that may be diagnosed, but not so clearly, without that aid.*

I will here take occasion to state, once for all, that only so much of the history, symptoms, and pathology of those diseases, and the constitution of the urine, will be noticed, as has direct reference to the object of this paper, which, is simply to urge the *value of the urine as a diagnostic agent*, without aiming to instruct in its practical application.

I. The first division embraces that interesting class of diseases usually denominated urinary; the most important, and those which will claim our attention, being diabetes (melletus), albumenuria, the various sedimentary and calculous affections, Bright's disease, and inflammation of the mucous membrane of the urinary passages.—Notwithstanding these diseases are termed urinary; yet it should be remembered, that all of them, with a few exceptions, are dependent for their existence upon some remote organic or functional affections. These primary affections, however, being mainly deduced from the state of the urine, and not presenting any other constant sign or symptom by which they may be known, have not, as yet, determined the *name*, though they may have suggested the treatment.

1. *Diabetes* is defined by Dr. Prout to be "a disease in which *a saccharine state of the urine* is the characteristic symptom."* Dr. Christison, on the other hand, denies that the mere presence of sugar is enough to constitute the disease, and adds—*with great tendency to emaciation and suppressed transpiration* † This author, however, admits that "the presence of sugar in the urine is by far the least variable of the phenomena which have been

* Prout on the Stomach and Renal Diseases. † Tweedie's Lib. of Prac. Med. Art. Diseases of the Urinary Organs.

hitherto carefully studied." As a definition can never express the exact nature and limits of a disease, but that definition being best, which answers the best practical ends, I can see no objection to Prout's. If sugar be found in the urine, every prudent practitioner would use those remedies adapted to diabetes, whether emaciation or dryness of skin be present or not; and if there be no sugar, of course the case could not be regarded as *diabetes*, unless the absence be only occasionally and temporary, and then its absence would in no way influence the diagnosis or treatment.

Various hypotheses have been offered, relative to the pathology of diabetes; but by far the most numerous and respectable authorities of the present day, refer the pathological *vice* to the assimilating functions, and not to the kidneys.

But as to the precise *seat* and *nature* of this vice, and the immediate source of the saccharine matter, authors do not fully agree; and perhaps will not, until further researches have more fully developed the laws of healthy and morbid actions.

Berzelius, (On the Kidneys and Urine,) considers the disease to "consist in a mal-assimilation of protein substances, from which, in healthy conditions of the body, urea is formed, but which in diabetes are converted from unknown causes, into sugar, ammonia, and, perhaps, an azotic extractive matter." Simon (Chemistry of Man,) has no doubt, from the extraordinary quantity of sugar excreted by those patients restricted to an animal diet, but *that the sugar is formed from the protein compounds.*—Dr. Maitland (Lond. Med. Gaz., May, 1840,) remarks that "the stomach, in diabetes, has the property of forming sugar from animal as well as from vegetable food." On the other hand, Dr. J. O. Rees (Manual on the Blood and Urine,) would seem to favor the opinion of the derivation of the sugar from saccharine and amylaceous food alone, and that the primary lesion may be found in the stomach. He gives considerable weight to the experiments of M. Mialhe, showing the power of the saliva, of converting starch into dextrine. Prout, it is well known, teaches that the sugar in diabetes is derived from a morbid state of the saccharine assimilating functions—that "the *reducing* function of the stomach is, for the most part, morbidly active, while the *converting** function is more or less suspended or paralyzed." He further suggests, that in the advanced stages of diabetes, sugar is derived from the gelatinous, and, perhaps, albuminous and oleaginous tissues, during the secondary or destructive assimilation. M. Bouchardat, (Braith. Ret. part xv.) whose observations have been extensive, has invariably found "that the quantity of sugar contained in the urine was always in a direct ratio with that of the bread or the feculent, or the saccharine food they had taken in the twenty-four hours." But amidst this want of agreement, amongst writers, as to whether or not nitrogenous matters may be converted into diabetic sugar there is scarcely any lack of agreement as to the more ready convertibility of sugar and fecula into that substance, and that therefore a knowledge of the saccharine nature of the urine is all-important to the diagnosis and treatment of the disease. Observations, too, have fully sustained the efficacy of the rational indications of treatment, for, observers most invariably agree as to the controlability of the quantity of glucose discharge, by confinement of the patient to an animal diet. Upon this subject, Dr. Rees remarks that "the general belief that diabetes is necessarily a fatal disease, has, in all probability, arisen from

* Dr. Prout, it will be remembered, teaches that saccharine principles, as starch,&c.,are converted, by primary assimilation, into albumin ous and oleaginous principles, and this is the *converting* function here alluded to.

the circumstance that it was formerly seldom detected until far advanced, and beyond the reach of remedies.—Attention having been of late, however, more actively directed to the general subject of urinary disease, cases are earlier detected and subjected to treatment; and it now occasionally happens that we meet with patients with a well authenticated and old diabetic history, who, on examination, show no signs of diabetes, appearing to have been permanently cured," &c. "If the amount of this saccharine or feculent food be diminished," says Bouchardat, "the proportion of urine excreted, and of sugar contained in it, will diminish in a corresponding degree. By suppressing, almost entirely, the use of this kind of food, the urine gradually becomes natural, both in quantity and composition."

2. *Albuminuria.* The next constitutional, or functional disease, to which I will advert, as depending upon the urine for diagnosis, is albuminuria. It was, some time after the researches of Dr. Bright, proving the co-existence of serous urine and certain organic changes in the kidney; before the profession began to regard albuminuria as a urinary disorder, apart from organic affections of the kidney; but observations have been so numerous on this point, as scarcely to leave an advocate for the identity of the two diseases. Prout says: "that in some individuals the urine is liable to become albuminous from certain derangements of the system in general, and of the kidneys in particular, which cannot at present be otherwise defined than as *functional* derangements." Dr. Graves (Lond. Med. Gaz., October. 1839,) contends that *the albuminous state of the urine is the cause of Bright's disease, and not the consequence.* Dr. Rees has been led to regard albuminuria as connected primarily with a degeneration of the blood, *the kidneys being the first link in the chain of causation, so far as the solids are concerned.*—Many other respectable authorities might be cited upon this point, but enough has been said to show that there is disease attended with an albuminous state of the urine, that cannot, with the present state of knowledge, be referred to any organic lesion, and is attended by no other constant sign or symptom, by which it may be recognized; and although it may be granted—as has been contended by some, and with great plausibility, too—that the kidneys are in a state of congestion or hyperemia, whenever albumen is found in the urine; yet as that congested state of the kidneys may be subsequent to a more general derangement, and in every such case, can only be assumed from the state of the urine, the diagnostic value of that secretion will not be lessened on that account.—But, allowing whatever disagreeament we may, as to the true pathology and proximate cause of albuminuria, there seems to be no disagreement as to its revealing a condition of the organism, that, if not promptly attended to, sooner or later terminates in renal degeneration, dropsy, and death—that it at least discloses a *dyscrasy*, or incipient constitutional depravity, that requires a careful protection against every species of exposure and intemperate living; and furthermore, that it is a disease by no means rare, and *can only be diagnosed from the state of the urine.*

3. *Urinary Sediments.*—The next subject that presents itself for notice under this head, relates to that group of diseases known as urinary sediments, sand, gravel, and calculi. These terms, however, having reference to the degree of accretion of these deposits, and not to any chemical or pathological distinction, has more of surgical than medical value, and will, therefore, not be applied in this paper.

As there are but few and trifling discrepancies of opinion, regarding the nature and origin of these deposits, and none with respect to their importance as signs of morbid action, I will briefly enumerate the most important and interesting of them, with a few remarks, touching their general history and pathology.

The sediments are various, and possess very different chemical constitutions, as they are derived from different principles, and different functional lesions in the economy. Some of them are merely the natural constituents of the healthy urine in excess, as urea,* uric acid, uric oxide, the urate of ammonia, and the earthy phosphates; whilst others are foreign to the healthy urine, as oxalate of lime, cystic oxide, and silicic acid, &c.

A. *Urea, uric acid*, and the *urates*, are, no doubt, derived from the proteine constituents of the blood; yet authors are not agreed as to their respective elemental origin, nor to the exact rationale of these formations.—Prout is of the opinion that urea is derived from the metamorphosis of gelatinous, and uric acid from that of the albuminous tissues, and Dr. Golding Bird (On Urinary Deposits) favors this view—at least, that they are derived from different tissues. Liebig, (Animal Chemistry,) on the other hand, supposes that uric acid is the immediate product of the metamorphic action, and that urea is a secondary product arising from the action of oxygen and water on the uric acid.† But these slight hypothetical differences, in no way impair the truth of the general statement, that they are derived from the effete and superfluous nitrogenous principles, and when found in positive excess in the urine, point to a source of disturbance of the functions of assimilation. that is all-important to diagnosis, and rational treatment.

B. *The Phosphatic Deposits*, are supposed, likewise, to

---

* *Urea* never occurs in sufficient quantity to form a deposit, yet from its relation to uric acid, and its salts, it is most convenient to consider it in this connection.

† Thus:—1 atom uric acid plus 4 atoms water plus 6 atoms oxygen are equal to 2 atoms urea plus 6 carb. acid.

be derived from the two sources of food and tissue. Much of the food of man has already formed within it some of the phosphatic salts, and the proteine elements of the tissues contain a given amount of phosphorus, which is readily converted into phosphoric acid by the action of oxygen; and the brain and nervous tissue, containing a larger amount of that substance than the other tissues, would, by hypothesis, yield a large share of the excess found in urine abounding in the phosphates; and observation fully sustains the hypothesis; for excessive mental and bodily exercise most invariably produce an excess of the phosphatic salts, and particularly the triple phosphates.

It is proper, also, to state that these salts are very often associated with serious organic lesions, and particularly of the spinal column; and the mucous membrane of the bladder when inflamed or highly irritated seems to have the power of secreting the phosphate of lime.

C. *Oxalate of Lime.* This deposit, as the result of a morbid state of the organism, was but little understood and appreciated before the researches of Dr. Golding Bird were published in the Medical Gazette, in 1842, and since, in his valuable monograph on urinary deposits.—Indeed, as an element of morbid urine, it was regarded as very rare; but that author has found it to be more constant than the earthy phosphates. Dr. Prout refers the constitutional cause of oxalic acid to a morbid action of the saccharine assimilating functions, and that it is closely related to diabetes melitus. Dr. Bird, however, has never found sugar in oxalic acid urine, nor oxalic acid in diabetic urine; nor has he found the general constitution of the two kinds of urine to agree. He therefore denies the saccharine and amylaceous origin of this acid. But from the constant association of oxalic acid with an excess of urea, and often with uric acid, as well as from the easy convertibility, by hypothesis, of urea and water—

minus one atom of oxygen—into oxalic acid and ammonia, Bird supposes that the acid in question is derived from the urea of the blood, and is associated with the uric acid diathesis.* He admits the correctness of Prout, in placing the primary cause in the digestive and assimilative functions; but refers the morbid derangement particularly to the functions of the stomach, duodenum, and liver.

D. *Cystine, or Cystic Oxide.* Since the discovery of this principle in a calculus, by Wollaston, a number of chemists have detected it in the urine, associated with calculi; but Dr. Robert Willis, I believe, was the first to detect it in the urine unconnected with stone. It is generally found in suspension in the urine, giving it a turbid appearance; but a portion, according to Bird, exists in a state of solution, as acetic acid always precipitates a small quantity.

Prout refers the pathology of this sediment to a morbid albuminous assimilation, and from the fatty matter often voided with the urine, as well as "the waxy character of the countenance," suggests the co-existence of *greasy liver.*

Bird coincides with Prout in referring the derivation of this substance to albumen. He thinks, from the chemical constitution of cystine, there can be no difficulty in explaining its presence in the urine, "by supposing that it is formed by those elements of our tissues which would normally have been converted into urea and uric acid, in consequence of the presence of an excess of sulphur, connected essentially with a scrofulous diathesis."

E. *Silicic Acid.* Berzelius and Simon being the only

---

* Liebig has also noticed the connection between the presence of the oxalates and urates in the urine, but he suggests that the oxalic acid is derived, along with the urea, from the oxidation of *uric acid.* —*Animal Chemistry.*

two chemists that I know of, who have detected silicic acid in healthy urine, and the latter only a *trace*, in the urine of a man, though *healthy*, "whose digestion and nutrition were not very good," I have thought proper to mention this as one of the ingredients foreign to healthy urine. As a deposit, but few credible examples are upon record. Upon the continent of Europe, this substance has been found, mixed with the earthy salts, in some three or four calculi.* In England, Drs. Yelloly and Venables† have each detected silica in notable quantities, and so blended with oxalate of lime as to satisfy the most sceptical against the idea of imposition. The proportion, however, of this substance, seems to have been hardly sufficient to justify the name of *siliceous gravel*. Dr. G. O. Rees is of the opinion that silica may be found in small quantities in most calculi, if it be made the special object of search. But be this as it may, it has been found in such small quantities and so seldom, that little or no pathological interest is attached to it, and is only noticed here, to render more complete the enumeration of urinary sediments.

4. *Morbus Brightii*. Of the organic diseases under this division, the first, in point of importance and interest, is Bright's disease of the kidney, sometimes known as granular degeneration of the kidney. This disease was discovered and made known by Dr. Richard Bright, of England, some twenty years ago, and the accuracy of his researches, and the originality of his discovery, have been fully confirmed by subsequent investigations.

No writer, of the present day, denies the existence of such a disease—often co-existing with albuminuria, and dropsy; most constantly fatal in its termination, and of

---

* By Vauquelin, Fourcroy, and Wurzer.
† See Braithwait's Retrospect, parts xiii. and xiv.

no unfrequent occurrence. But the point, it seems, that has been most difficult to decide in its history, is the nature of the relation of the organic affection to the albuminous state of the urine; and this is the point which most directly interests us in establishing the value of the urine as the medium of its diagnosis.

Having already shown that albuminous urine does not necessarily imply the presence of Bright's disease, it only remains to be inquired whether Bright's disease is always associated with albuminous urine; and as authors are pretty generally agreed upon the affirmative of this question, I will only notice those facts and opinions, at present known to me, that may seem to militate against this doctrine.

Simon, who always found albuminous urine in this disease, gives Prof. Graves, of Dublin, the credit of denying its constant presence; but when an examination is made of the recorded postmortem of this case, with "Bright's kidneys" secreting "perfectly healthy urine," it will be found that Dr. Graves's denial amounts to nothing. The appearance of the kidneys is thus described: "This man's kidneys were found somewhat enlarged, and at first I thought they were otherwise natural, but a further examination and maceration convinced us that the corticle substance was paler than usual. Increase of size, paleness, and, *perhaps*, a very slight softness of the cortical part, constituted the whole change; there was no appearance of granulations either on the surface, or in the substance of the kidneys."* Besides, this man had had chronic dropsy, with highly albuminous urine, but as the dropsy disappeared, the urine gradually became healthy, and remained so a fortnight, when he took erysipelas

* See Lecture 2nd, of Dr. Graves, at Meath Hospital, session 1837—8, and published in Lond. Med. Gaz., October, 1838.

of the face (the urine again becoming albuminous) and died. Now this case, it seems to me, is not difficult of explanation. It appears he labored under dropsy with albuminuria, which is of frequent occurrence; the return of albuminuria on the acute attack, may be referred to engorgement of the kidney. The postmortem really disclosed nothing but hypertrophy. The *very slight softening*, from Dr. G.'s own showing, was very questionable, and the history of the case would well account for the paleness of the kidney, on the ground of general *anemia.* And, furthermore—and what seems a little inconsistent—in a subsequent part of this lecture, Dr. Graves is willing to admit, that *a well characterized* case of Bright's kidney, existing without albuminous urine, *has never been observed.* Dr. Christison, who has paid much attention to this malady, says that albumen is always present in the acute stage, but sometimes absent in the chronic form; and there are other evidences of its occasional absence in the more advanced stages.

Admitting this to be true, and the practical diagnostic value of the urine will be but little impaired, since that stage in which it disappears can scarcely be remedied by treatment.

Dr. Watson (Lectures on Practice of Med.) declares that albumen is not always present in Bright's disease—but he only alludes to its occasionally vanishing a few hours, after a hot air bath, active purging, &c.; which amounts to no denial of its *constant general presence.* Indeed I want no better advocate for the diagnostic value of the urine, in this disease, than Watson, who says:—" Now it is worth your while to remark, with respect to this category of symptoms that (the state of the urine excepted) they have no special prima facie reference to renal disease, they are all common enough in other complaints.

"In truth, they are mere secondary consequences of

Bright's disease; and in so far as they are symptoms of it, they are indirect symptoms. Before Dr. Bright, no one perceived in such symptoms any indication of disease of the kidney. The primary and fundamental organic malady reveals itself by no direct symptoms except those which are furnished by the urine.

5. *Inflammation of the Urinary Passages.* Inflammation of the mucous membrane of the urinary passages, may be suspected from certain painful symptoms in the direction of those organs, febrile action, &c.; but it cannot be positively diagnosed (except of the urethra) without the information furnished by the urine. The presence of mucus and epithelium scales is almost pathnomonic of this affection. In the chronic form, phosphate of lime usually co-exists; and pus, both in the chronic and acute stage may be present. Dr. Venables* "once saw an instance in which, during the acute stage of inflammation, enormous quantities of pus were secreted and thrown off by the mucous coat of the bladder, similar to what takes place in acute bronchitis." You have now presented to your view, some of the more important of a large class of diseases, that are of very frequent occurrence in this and every other country—none of which can be discovered and identified without the information furnished by the physico-chemical constitution of the urine. And I now proceed to notice, hastily,

II. *Those diseases that may be diagnosed, but not so clearly, without the aid of the urine.* In truth, the condition of the urine may throw some light upon the diagnosis of nearly every disease that may afflict the human body; but it will fall within the object of this paper to notice only such as have a more immediate and constant patho-

---

* Lectures on Calculus and Urinary Disorders—Lond. Med. Gaz. for August, 1839.

logical relation with that fluid, and even these are too numerous to admit of more than a general and hasty consideration, in a paper aiming more to call attention to facts, than to elucidate their nature.

1. *Disorders of the Brain and Nervous System;* as coma, convulsions, hysteria, nervous exhaustion, &c.

Notwithstanding these diseases, as already stated, may be diagnosed without reference to the urine; yet, that secretion, it is allowed by every one, throws much light on their peculiar nature, and at times, may become absolutely essential to a correct diagnosis.

*Coma*, and certain forms of *convulsion*, at times, owe their origin to a deficient excretion of urea, and, consequently, its accumulation in the blood; whilst at other times, they proceed from very different causes, as narcotic poisons, indigestible substances in the stomach and bowels, intestinal worms, cerebral congestion, hysteria, &c.; and hence it cannot but be obvious that the renal secretion is no unimportant agent in determining the nature of such cases.

*Hysteria* is another diseases that is pretty generally associated with a characteristic state of the urine. It is usually abundant and of low specific gravity. That hysteria, may, under most circumstances, be diagnosed very readily, will not be denied, yet it sometimes so assimilates other diseases, as epilepsy, tetanus, &c., that a satisfactory diagnosis is quite difficult.

*Nervous Exhaustion*, or that disordered state of the nervous system usually denominated *nervousness*—where great mental exercise or anxiety has caused considerable waste of the nervous tissue,—the kidneys, as in hysteria, often secrete a quantity of pale limpid urine; but differs from hysterical urine in containing usually an excess of the ammoniaco-magnesian phosphates.

2. *Chronic Organic Diseases*, as of the heart, liver, spleen, &c., are well known to produce certain changes in the secretion of the kidneys—such, however, as will not be contended, are peculiar to these diseases—yet when accompanied by other symptoms, go very far in elucidating their nature. Thus, in auriculo-ventricular contraction of the heart, and in certain lesions of the liver and spleen, as cirrosus, and induration of the spleen, the urine is secreted in small quantities, of high specific gravity, abounding in sediments, and deeply colored by the presence of purpurine.

Hypertrophy of the heart, too, is often associated with an albuminous urine.

3. *Chronic Functional Diseases*, as dyspepsia, or indigestion, &c.

The terms dyspepsia and indigestion are exceedingly latitudinous, and indefinite in their meaning. They embrace a large number of disorders, having no common resemblance, except defective or vitiated nutrition. The primary and most important seat of the disorder may be found in every series of the assimilating functions, from the digestive actions in the stomach to the destructive processes in the peripheral vascular system; and in each series it may have several independent sources. According to Prout, and I believe his classifications in the general are adopted by the profession, indigestion may be referred to a pathological state of the functions of aqueous, saccharine, albuminous, and oleaginous assimilations—and these pathological departures may be found in different stages of these processes of assimilation. And hence the importance of a more specific diagnosis, than that which merely arrives at the indefinite notion of *indigestion*, or *dyspepsia*. I have already shown the *general* relations of the assimilating functions to the renal secretion, and also that in certain *special* derangements of these functions, certain more or less constant alterations may be found in the urine, as excess of urea, uric acid and urates, the presence of oxalic acid, cystine, sugar, &c. Then, when dyspeptic symptoms make their appearance, it is clear that the urine alone can furnish that clue to the precise nature of the complaint, that is requisite to a rational and safe treatment.

4. *Acute Inflammatory Diseases.* The last group of diseases, that will be noticed, are some of the acute inflammatory diseases, as gout, rheumatism, acute dropsy, nephritis, &c. It is well known that each of the diseases here mentioned, is sometimes liable to be confounded with others, and it is equally well known that each is usually associated with a certain morbid alteration of the urine. Thus, gout and rheumatism were attended with a high colored urine—often abundant, and loaded with sediments of uric acid and the urates of ammonia and soda; acute dropsy, with urine of sparse secretion, albuminous, and high specific gravity. The urine in nephritis may be variable, according as the investing capsule, cortical, and tubular portion, or the pelvis of the kidney may be the seat of inflammatory action. In these affections, it may vary but little from a healthy state, as in *peri-nephritis*—albuminous, as in *inflammation of the cortical and tubular portion*—or contain mucus and pus, as in *pyelitis.*

I will now conclude by briefly mentioning the late supposed discovery of a specific constitution of the urine in *Pregnancy*, which, though not a disease in itself, may often be confounded with diseases; and, besides, the diagnosis of pregnancy is sometimes of great value and interest in a medico-legal point of view. The principle that has been supposed to characterize the urine in gestation has received, from its discoverer, [Nanche] the name of *kystein,* or *kiestein.* Many experiments have been made to ascertain the truth and value of this discovery, and although kiestein has not been found invariably in pregnancy—and it is even rendered probable that it may occur under other circumstances, as in lactation (Kane)—yet they nearly all concur in proving its presence in the urine, as a general thing, sometime in the course of gestation.

This state of the urine, however, is not, at present, regarded as depending upon the presence of any *new simple substance;* but to owe its peculiarity to the admixture of a *proteine substance* resembling caseine, a *butyraceous fat*, and the *ammoniaco-magnesian* phosphates. But this, even of a *proximate elementary character* does not render it the less valuable as an agent in diagnosis, if it be found to exist *only under certain conditions.* Without detaining you with the details of experiments—regarding the diagnostic value of kiestein, Simon (Chemistry of Man) remarks: "From the observations of Kane and myself, it seems to follow, that pregnancy may exist without the occurrence of kystein in the urine; if, however, there is a probability or possibility of pregnancy, and kystein is found in the urine, then the probability is reduced almost to a certainty." Upon the same subject, Dr. Bird (Urinary Deposits) concludes, from his observations: "As a test for the existence of pregnancy, the formation of the caseous pellicle, especially if accompanied by a cheese-like odor, will be an extremely valuable *corroborative* indication: but it would be unsafe to found on it alone any positive opinion, because, as a sufficient number of observations have not yet been made on this subject, we have no right to assume, however probable it may be, that a caseous pellicle can appear *only* when pregnancy exists."

Since this paper has been ordered for publication—to give it as much *practical* value as possible—I have constructed the following table, to assist such as may wish to avail themselves of the helps furnished by morbid urine, in the difficult task of diagnosis; and who may not have the advantage of a competent chemical education. It will be observed that the instruments and reagents necessary for all the manipulations in the Table, are *few, simple, and easy of application.*

### A TABLE TO ASSIST THE BEGINNER IN THE DIAGNOSTIC APPLICATION OF MORBID URINE.

| SUBSTANCE. | PATHOLOGICAL RELATIONS. | CHARACTER OF URINE | CHARACTER OF DEPOSIT. | MANNER OF TESTING, &C. |
|---|---|---|---|---|
| UREA. | Found in excess sometimes in inflammatory affections, in a certain form of di uresis called by Willis, Azoturea, in oxalic acid, urine, &c. Deficient in diabetis insipidus, hysterical diuresis, &c. | Color and other sensible qualities variable ; specific gravity, high ; acid reaction. | Being soluble in an equal quantity of cold, and every proportion of warm water, there is no deposit. | Put a small portion of the suspected urine in a watch-glass, add an equal quantity of nitric acid, and if urea be in excess, crystals of nitrate of urea will immediately form. To determine whether the excess be absolute or relative, the amount of urine discharged in the twenty-four hours should be ascertained. |
| URIC ACID. | In excess, in inflammatory, and some febrile affections, gout and rheumatism, certain diseases of the lungs, liver, and heart, indigestion, &c. | Color high ; S. Gr. slightly increased; acid; often associated with the urates, and *purpurine*. | Chrystaline and red, from the coloring matter of the urine. | Insoluble in water, readily soluble in caustic potash, and precipitated from this menstruum by the addition of an acid ; in a granular and colorless state, dissolved by nitric acid with effervescence, and by careful evaporation to dryness, yields a pink color, which becomes a fine violet by the action of ammonia. |
| URATE OF AMMONIA. | Pathological relations much the same as uric acid.—Urate of *soda* present in gout and rheumatism. | Color varies from pale to deep copper; S. Gr. variable; often low; acid; becomes turbid on cooling. | Varies from white to purple ; pink or fawn color most common ; amorphous. | Subject a small portion of the turbid urine in a test tube, to a gentle heat from the blaze of a spirit lamp, when the urates will dissolve, rendering the urine perfectly clear. It will be well to watch the process of clearing, as albumen, if present, will render the specimen again turbid, at a higher heat. |

| SUB-STANCE | PATHOLOGICAL RELATIONS. | CHARACTER OF URINE | CHARACTER OF DEPOSIT. | MANNER OF TESTING, &C. |
|---|---|---|---|---|
| PHOSPHATES. | Nervous exhaustion in excess; diseases and injuries of the spinal column; marasmus; abuse of the sexual organs; mercurial cachexia; certain periods of continued fever; inflammation and irritation of the bladder, &c. | Color variable, often pale; Sp. Gr. variable, mostly low; alkaline; triple phosphate often acid; opulescent. | White, except colored with blood; phosphate of lime amorphous; triple phosphate; chrystaline. | Soluble in dilute hydrochloride and nitric, and strong acetic acids; insoluble in ammonia and liq. potass; heat does not dissolve; mucus and pus often present; and sometimes blood; triple phosphate may often be detected on the surface of urine that has stood for a time, forming a thin irridescent pellicle, or sheet of minute crystals. |
| OXALATE OF LIME. | Associated with chronic and peristent derangement of the general health; dispepsia, mercurial and syphilitic cachexia; exhaustion from child bearing, and over lactation; venerial excesses, &c.—(Bird) | Pale citron yellow, or greenish hue; (Prout.) fine amber hue; often darker than natural; (Bird.) Sp. Gr. variable; acid. | A white, glistening powder diffused through the urine, but does not fall as a distinct deposit. (Bird.) | Insoluble in aqua potass, and acetic acid; soluble in nitric acid, and convertible by a red heat into carbonate of lime. The microscope reveals beautifully formed transparent octohedral crystrals, with sharply defined edges and angles, &c.—(Bird.) |
| CYSTIC OXIDE. | But little seems to be known; Prout suggests its connexion with malignant disease, and particularly with *fatty liver*. Bird is of opinion that it is associated with the scrofulous diathesis. | Greenish or honey yellow; sometimes oil on the top; odor of sweet-briar; Sp. Gr. generally low; neutral, or slightly acid. | White or fawn colored pulverulent deposit, much resembling the pale variety of urate of ammonia.—(Bird.) | Heat does not dissolve it as it does urate of ammonia; nor dilute hydrochloride, and strong acetic acids, as they do the earthy phosphates; readily soluble in liquor ammonia; microscope reveals crystals in the form of six sided laminæ. |

| SUBSTANCE. | PATHOLOGICAL RELATIONS. | CHARACTER OF URINE | CHARACTER OF DEPOSIT. | MANNER OF TESTING, &C. |
|---|---|---|---|---|
| SUGAR. | The presence of sugar in the urine is pathognomonic of diabetes mellitus, which has for its most usual associates ,certain forms of indigestion ; boils and carbuncles, &c.—In the latter stages,phthisis; jaundice ; dropsy ; apoplexy, &c.—(Prout.) | Pale straw color; clear; odor of hay; sweetish taste; Sp. Gr. high; acid reaction. | | There are many tests for diabetic sugar, but the following, (Moore's test,) from its easy application, and, according to Bird, from its remarkable freedom from sources of fallacy, may suffice here. Boil for a minute or two in a test tube, two or three fluid drachms of the urine with half the quantity of liquor potassæ, and if sugar be present, the urine will become of an orange yellow, brown or claret color, by conversion of the sugar into melassic acid ; the *yeast test,* and also Trommer's, might be used as confirmatory. |
| ALBUMEN. | Found in the urine, in Bright's disease of the kidney; albuminuria ; certain forms of chronic and acute dropsy ; and other chronic affections; the latter stages of acute exanthemata; gout ; rheumatism, &c. | Sensible qualities, density &c.; variable; if albumen be abundant it has a sticky feeling. | | Heat a portion of the urine, in a test tube, to the boiling point, and if it becomes turbid, the precipitate is either albumen, or earthy phosphates ; if the latter, a drop or two of nitric acid will clear it again, and if the former, it continues turbid ; if the urine be alkaline, add a few drops of nitric acid before heating ; if in this case the patient has been using balsam or cubebs, an equal quantity of alcohol should be added to the turbid urine, which again clears, if the deposit be not albumen. |

| SUBSTANCE. | PATHOLOGICAL RELATIONS. | CHARACTER OF URINE. | CHARACTER OF DEPOSIT. | MANNER OF TESTING, &C. |
|---|---|---|---|---|
| MUCUS. | In excess, in inflammation and irritation of the genito-urinary passages; from various causes, as cold; mechanical irritation of calculi, &c. | Color, &c., variable; cloudy; sometimes gelatinous; Sp. Gr. variable; generally alkaline. | Stringy or ropy masses. | Add acetic acid, and if the deposit be mucus it "coagulates into a thin semi-opaque corrugated membrane, presenting an appearance so peculiar, that once seen it can never be mistaken." (Bird.) |
| PUS. | Presence in the urine indicates, suppuration of the kidneys, or mucous membrane of the genito-urinary passages, abcess in adjoining parts, &c. | Color, Sp. Gr., &c. variable; turbid; acid or neutral. | A dense homogeneous layer of a pale greenish cream color, uniformly diffusing on agitation. | Agitate a portion of the deposited pus with an equal quantity of liquor potassæ, and a dense translucent, gelatinous or mucous mass forms; by subjecting the supernatant urine to the test of heat and nitric acid, albumen may be detected; not coagulated by acetic acid as mucus, and undergoes putrefaction slowly. |

